# Primary tracheal small cell carcinoma: A case report and discussion of its surgical and oncological management

**DOI:** 10.1016/j.xjtc.2025.09.001

**Published:** 2025-09-10

**Authors:** Shantel Chang, Caroline Cooper, Wingchi Lo

**Affiliations:** a Departments of Cardiothoracic Surgery, Princess Alexandra Hospital, Brisbane, Queensland, Australia; b School of Medicine and Dentistry, Griffith University, Gold Coast, Queensland, Australia; c Departments of Anatomical Pathology, Pathology Queensland, Princess Alexandra Hospital, Brisbane, Queensland, Australia; d School of Medicine, The University of Queensland, Brisbane, Queensland, Australia

**Figure:**
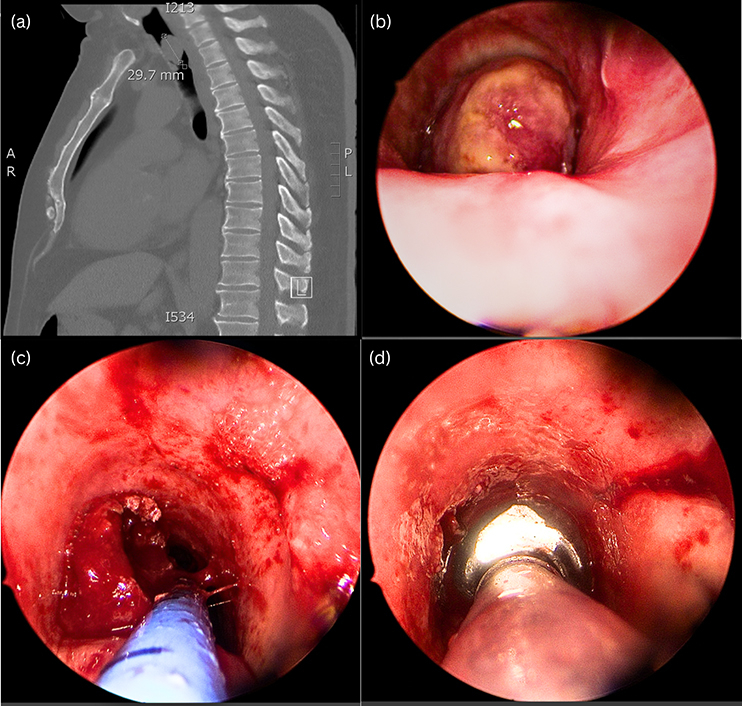
Primary small cell carcinoma presenting in near-occlusion of the trachea.

**Central Message**

Primary tracheal small cell carcinoma is a rare and frequently misdiagnosed malignancy. This case reports endotracheal resection, with combined chemoradiotherapy and immunotherapy.

Primary tracheal malignancy represents 0.2% of respiratory malignancies and 0.04% of all cancers.^1^ Of these, squamous cell carcinoma, followed by adenoid cystic carcinoma, are the most predominant histology of tracheal tumors.^1^ Primary neuroendocrine carcinoma of the trachea is extremely rare, with the literature limited to a handful of case reports or epidemiological studies.^2^ As such, there is frequent misdiagnosis and no clear guidelines on management. We report a case of primary tracheal small cell carcinoma and its management. Written informed consent was obtained and ethical review exempted (EX/2025/QMS/118480) on May 21, 2025.

## Case Report

A 65-year-old man presented to the emergency department with a 3-month history of dyspnea and nonproductive cough. He had a history of asthma and sought medical attention from his general practitioner, who had treated the patient for an infective exacerbation of asthma with courses of antibiotics and high-dose prednisolone, without effect.

**Figure 1:**
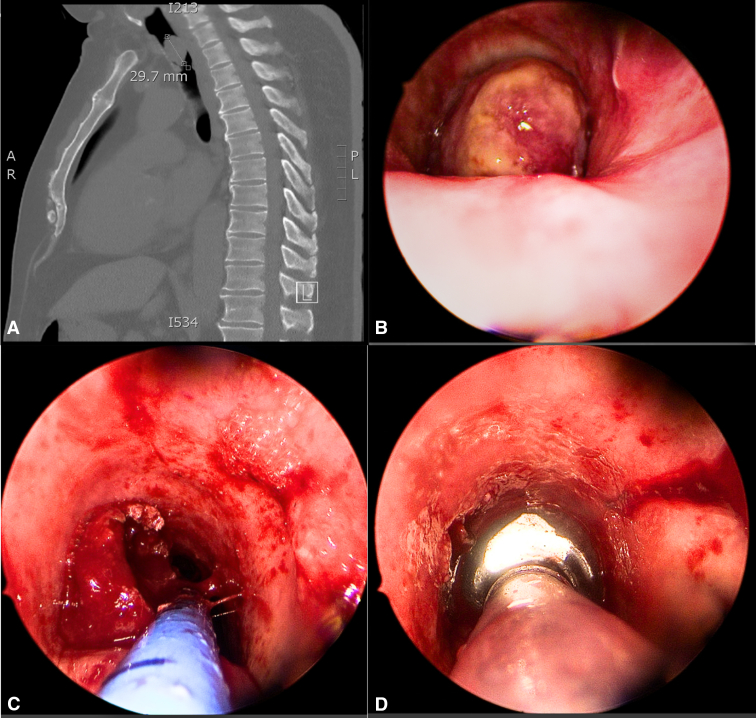
A, Preoperative computed tomography. B, Intraoperative view of tracheal lesion. C, During resection with coblation. D, After resection.

The patient's background was significant for hypertension, dyslipidemia, obesity, and type 2 diabetes mellitus. Although he was a nonsmoker, he had passive smoking exposure of 45 pack-years from another household member. There was no family history of malignancy or environmental exposure to respiratory carcinogens. On examination, there was no stridor, oxygen requirement, or increased work of breathing. He had no signs suggestive of cardiogenic dyspnea. Computed tomography showed a subglottic 20 × 16 × 29 mm tracheal nodule, causing near-complete occlusion of the trachea (Figure 1). A positron-emission tomography scan showed no nodal or distant metastasis.

He underwent urgent suspension laryngo-bronchoscopy with endotracheal debulking of the lesion. Spontaneous ventilation in sedation was first adopted, with oxygenation through a Cook's catheter passed around the lesion. Exposure was achieved with a Lindholm laryngoscope (Figure 1). The lesion arose from the midtrachea, in the 8 o'clock position, occluding 90% of the lumen. The lesion was debulked with a coblation device, and biopsies were taken under laryngoscopic vision. After resection, intubation was completed with a microlaryngeal endotracheal tube and flexible bronchoscopy was used to suction the airway distally. After resection, there was no macroscopic tumor remaining (R1 resection). The patient was transferred to the intensive care unit and extubated the following day after intravenous dexamethasone to prevent airway edema.

**Figure 2:**
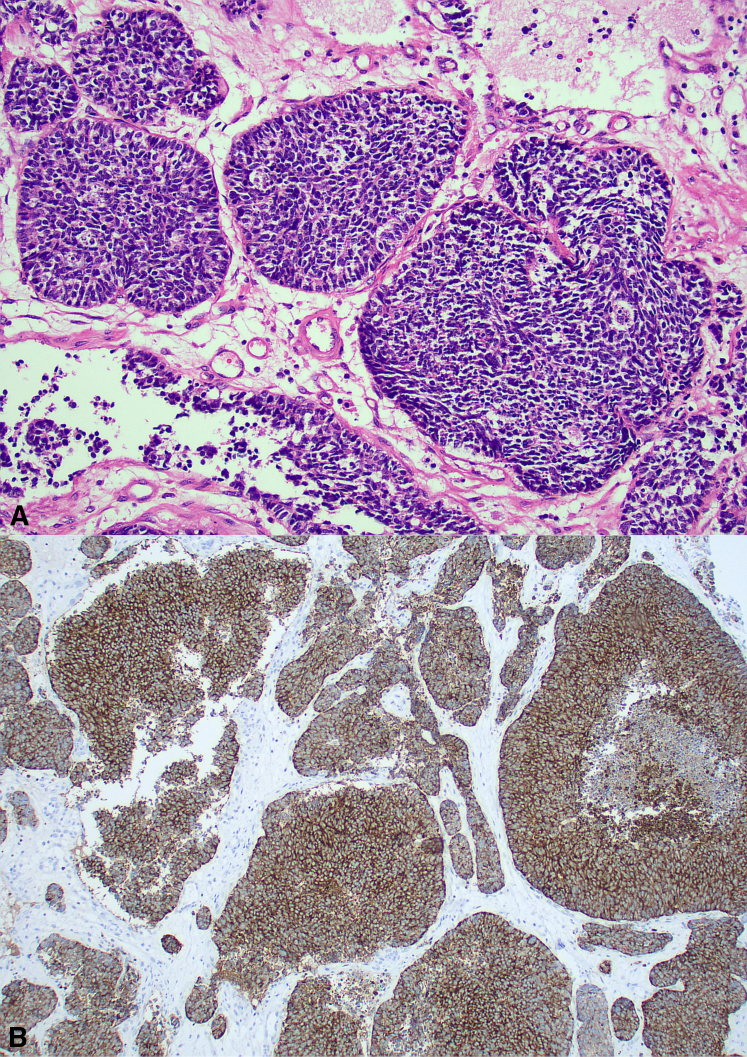
A, Hematoxylin and eosin-stained section showing typical morphology of small cell carcinoma (×200). B, Synaptophysin-stained section confirming neuroendocrine differentiation (×100).

Histopathology confirmed small cell carcinoma, with cytokeratin AE 1/3, insulinoma-associated-protein-1, and synaptophysin positivity on immunohistochemical staining (Figure 2). Thyroid transcription factor 1 showed patchy staining and p40 was negative. He completed chemoradiotherapy with cisplatin and etoposide over 4 cycles and external beam radiotherapy, which he tolerated well. Following chemoradiotherapy, he is planned for consolidation immunotherapy with durvalumab. He has minimal morbidity and no radiological recurrence at 6 months postoperatively.

## Discussion

Therapeutic strategies for treating tracheal small cell carcinoma are based on historical treatments and extrapolated from treating small cell carcinoma of the lung. There are limited reports on resection of tracheal small cell carcinoma, with reports of tracheal stenting and limited biopsy, resulting in poor outcomes.^3^^,^^4^ Heikal and colleagues^4^ report endobronchial debulking and chemotherapy resulting in increasing size of the lesion at 12 months and death from pulmonary embolism and respiratory failure. Qiu and colleagues^3^ report tracheal stenting and chemoradiotherapy with eventual death from massive hemoptysis. The role of surgical resection, whether it be complete, nonradical, or palliative, remains controversial. Studies have shown that resection was associated with longer survival, although significance was variable.^5^ Radical surgery with tracheal resection and primary anastomosis, although achieving a more complete oncological resection, exposes the patient to invasive surgery and its associated surgical risk. To date, there are no studies comparing endoscopic, radical, and nonradical resection due to the rarity of the cancer.

The systemic regimen typically involves cisplatin and etoposide with concurrent radiotherapy.^5^ In recent years, combination therapy with the immune checkpoint inhibitors have yielded improved survival, which advocates for this combination as first-line therapy.^2^ In conclusion, this case reports safe endotracheal surgical resection of primary tracheal small cell carcinoma and implementation of first-line systemic treatment, with minimal morbidity at 6 months’ follow-up.

## Conflict of Interest Statement

The authors reported no conflicts of interest.

The *Journal* policy requires editors and reviewers to disclose conflicts of interest and to decline handling or reviewing manuscripts for which they may have a conflict of interest. The editors and reviewers of this article have no conflicts of interest.

## References

[1] Webb B.D., Walsh G., Roberts D., Sturgis E. (2006). Primary tracheal malignant neoplasms: the University of Texas MD Anderson Cancer Center experience. J Am Coll Surg.

[2] Chen K., Yang Z., Zhang X. (2020). Clinical features and prognosis of primary tracheal small cell carcinoma: a population-based analysis. Transl Cancer Res.

[3] Qiu J., Lin W., Zhou M.L., Zhou S.H., Wang Q.Y., Bao Y.Y. (2015). Primary small cell cancer of cervical trachea: a case report and literature review. Int J Clin Exp Pathol.

[4] Heikal M. (2012). Small-cell cancer presenting as a tracheal polyp. J Bronchology Interv Pulmonol.

[5] Chen Y., Zhu H., Wang D., Ye Y., Gao J. (2024). A novel perspective on the treatment of primary tracheal small cell carcinoma: a patient’s experience with immuno-combined EP therapy and literature review. Front Immunol.

